# Extraction of soybean plant trait parameters based on SfM-MVS algorithm combined with GRNN

**DOI:** 10.3389/fpls.2023.1181322

**Published:** 2023-07-25

**Authors:** Wei He, Zhihao Ye, Mingshuang Li, Yulu Yan, Wei Lu, Guangnan Xing

**Affiliations:** ^1^ College of Engineering, Nanjing Agricultural University, Nanjing, China; ^2^ Soybean Research Institute, Ministry of Agriculture and Rural Affairs (MARA) National Center for Soybean Improvement, Ministry of Agriculture and Rural Affairs (MARA) Key Laboratory of Biology and Genetic Improvement of Soybean, National Key Laboratory for Crop Genetics & Germplasm Enhancement and Utilization, Jiangsu Collaborative Innovation Center for Modern Crop Production, College of Agriculture, Nanjing Agricultural University, Nanjing, China; ^3^ College of Artificial Intelligence, Nanjing Agricultural University, Nanjing, China

**Keywords:** structure from motion, soybean plant, 3D point cloud, plant phenotype, 3D trait extraction

## Abstract

Soybean is an important grain and oil crop worldwide and is rich in nutritional value. Phenotypic morphology plays an important role in the selection and breeding of excellent soybean varieties to achieve high yield. Nowadays, the mainstream manual phenotypic measurement has some problems such as strong subjectivity, high labor intensity and slow speed. To address the problems, a three-dimensional (3D) reconstruction method for soybean plants based on structure from motion (SFM) was proposed. First, the 3D point cloud of a soybean plant was reconstructed from multi-view images obtained by a smartphone based on the SFM algorithm. Second, low-pass filtering, Gaussian filtering, Ordinary Least Square (OLS) plane fitting, and Laplacian smoothing were used in fusion to automatically segment point cloud data, such as individual plants, stems, and leaves. Finally, Eleven morphological traits, such as plant height, minimum bounding box volume per plant, leaf projection area, leaf projection length and width, and leaf tilt information, were accurately and nondestructively measured by the proposed an algorithm for leaf phenotype measurement (LPM). Moreover, Support Vector Machine (SVM), Back Propagation Neural Network (BP), and Back Propagation Neural Network (GRNN) prediction models were established to predict and identify soybean plant varieties. The results indicated that, compared with the manual measurement, the root mean square error (RMSE) of plant height, leaf length, and leaf width were 0.9997, 0.2357, and 0.2666 cm, and the mean absolute percentage error (MAPE) were 2.7013%, 1.4706%, and 1.8669%, and the coefficients of determination (R2) were 0.9775, 0.9785, and 0.9487, respectively. The accuracy of predicting plant species according to the six leaf parameters was highest when using GRNN, reaching 0.9211, and the RMSE was 18.3263. Based on the phenotypic traits of plants, the differences between C3, 47-6 and W82 soybeans were analyzed genetically, and because C3 was an insect-resistant line, the trait parametes (minimum box volume per plant, number of leaves, minimum size of single leaf box, leaf projection area).The results show that the proposed method can effectively extract the 3D phenotypic structure information of soybean plants and leaves without loss which has the potential using ability in other plants with dense leaves.

## 1 Introduction

Soybean is an important grain and oil crop worldwide and is rich in high-quality protein, unsaturated fatty acids, isoflavones, and other nutrients (Zhang T et al., 2019). The phenotypic morphological characteristics embodied in the growth process play an important role in the selection of excellent soybean varieties (Zhu et al., 2020), and the phenotypic state of plants is the physical manifestation of the genotype (Alonge et al., 2020), which is not only of great significance for the quantitative analysis of genotype-environment interactions (Barker et al., 2019; Van Eeuwijk et al., 2019), but also for breeding activities, such as optimal cultivation, fertilization, and irrigation of plants (Chawade et al., 2019; Li et al., 2021). Phenotypes are prone to changes in response to genetic mutations and environmental influences (Vogt, 2021), which are the main bottlenecks limiting the expansion of genomics in plant sciences, animal biology, and medicine. Different genes determine different insect resistance in plants, affecting plant phenotypes (Tyagi et al., 2020). Therefore, accurate and non-destructive acquisition of soybean phenotypic parameters is essential for the study of soybean plants and breeding of insect-resistant varieties.

Chen et al. (2021). constructed the 3D model of soybean plant can efficiently obtain its geometric characteristics and morphological traits, which is essential for understanding plant growth and plant response to biotic and abiotic stresses, so as to estimate the growth rate of soybean plants and predict the tolerance of stress, it greatly reduces the marginal cost of collecting multiple morphological traits across multiple time points, which has important theoretical significance and practical value for soybean variety selection and breeding, scientific cultivation and fine management (Wang et al., 2022). By means of the 3D model of the plant, the growth situation and specific changes of the plant can be quickly understood, which contributes to screen out excellent varieties with high quality and strong insect resistance, and can also lay the foundation for the genetic improvement of soybean and breed better varieties (Xue et al., 2023).

The traditional methods used to obtain plant phenotypic parameters include manual measurement, two-dimensional (2D) image measurements, and precision instrument measurements. Manual measurements are slow, costly, and subjectively inaccurate (Gage et al., 2019), which can easily damage plants during measurement. When plant phenotypic parameters are measured based on 2D image technology (Das Choudhury et al., 2020; Li et al., 2020; Omari et al., 2020; Kuett et al., 2022), critical spatial and volumetric information, such as thickness, bending, and orientation, is easily lost during data conversion from three-dimensional (3D) to 2D states, and the morphology will also be blocked from different perspectives (Martinez-Guanter et al., 2019). Precision instruments, such as handheld laser scanners (Artec EVA laser scanners and FastSCAN laser scanners) (Ma et al., 2022), 3D laser scanning, and radar technology (FARO Focus3D 120 laser scanning of ground objects) (Junttila et al., 2021; Nguyen et al., 2022), are often used to measure plant phenotypic traits. Although it has a high resolution and can reconstruct the 3D model of the plant with high precision and record the phenotypic information of the plant (Ao et al., 2022), its acquisition speed is slow, the equipment is expensive, and the lack of color information for the obscured parts of plants fails to accurately reflect phenotypic traits. In addition, for automatic analysis of plant phenotypic information, 3D point clouds generated by laser scanners must be correctly extracted from a large amount of 3D data and classified for this purpose. The high cost and limited availability of laser-scanning equipment hinder its wide applications.

Recently, scholars have been increasingly interested in the structure from motion (SFM) algorithm based on multi-view stereo measurement, and a series of exploratory studies have been carried out in the fields of geographical environment and agriculture. The 3D model can be automatically reconstructed according to overlapping 2D digital image sets (Jiang et al., 2020), which has the advantages of being self-calibrated, less constrained by the environment, and functional both indoors and outdoors, and has been widely used in 3D reconstruction (James et al., 2019; Swinfield et al., 2019). Ewertowski et al. (2019) used UAV combined with this technology to quickly and ultra-high-resolution 3D reconstruction of glacier landforms, and drew the terrain related to glaciers in detail. In the field of agriculture, He et al. (2017) used this technology to obtain 3D models of strawberries and used custom software to process point cloud data and obtain seven agronomic traits of strawberries. Huang et al. (2022) used the DoidiltenGAN image enhancement algorithm combined with SFM-MVS algorithm to develop a set of agricultural equipment that could accurately perceive the growth of crops under low light. Hui et al. (2018) used this technology to obtain 3D point clouds for cucumbers with flat leaves, peppers with small leaves, and eggplants with curly leaves. With the help of precision instruments and Geomagic Studio software, they measured five characteristic parameters of the plant, including leaf length, leaf width, and leaf area, and analyzed the errors between them. In (Xu et al., 2019), a UAV was used in combination with this technology to obtain a 3D model of cotton, and a DEM was used to measure four phenotypic traits, such as plant height and canopy coverage. In (Piermattei et al., 2019), this technology was used to obtain 3D point clouds of trees and four parameters, such as DBH and the number of trees. With the rising demand for different types of phenotypic information from 3D point clouds, Rahman et al. (2017) explored future research on volume measurement and modeling using this method to obtain 3D models.

These studies show that the SFM algorithm has good potential in the field of plant phenotype detection. However, at present, the analysis of phenotypic trait parameters of plants is limited, most software is used, and there is a lack of technology for reconstruction and phenotype measurement of plants with various and dense leaves. Therefore, in this study, we combined structure from motion (SFM) with multiple view stereo (MVS) methods to build a platform for acquiring plant sequence images. Using the soybean seedlings with different gene expression patterns of the same soybean plant at the R4 stage as the research object, the point cloud models were obtained by 3D reconstruction using different sequence images, the LPM algorithm was used to quickly perform non-destructive phenotype measurements, and the accuracy of phenotype measurement was evaluated. The feasibility of SFM-MVS technology combined with the LPM algorithm is explored and the phenotype and insect resistance of soybean plants are analyzed.

At present, machine learning (ML) and deep learning (DL) algorithms are widely used in the plant phenotype classification. For machine learning (ML), Tan et al. (2021) used the machine learning (ML), based on tomato cultivation as well as disease datasets to classify plant diseases; Barradas et al. (2021) applied different machine learning (ML) methods such as Decision Tree (DT), Random Forest (RF), and Extreme Gradient Boosting (XGBoost) to classify plants into three drought stress levels; Alam et al. (2020) used random forests (RF) for detection and classification of weeds as well as crops and accurate identification and control of weeds. For deep learning (DL), Ferentinos et al. (2018). made use of Convolutional Neural Networks (CNN) to classify plant disease images; Brugger (2022). analyzed spectral data of plant phenotypes based on deep learning (DL) to forecast plant diseases and categories; Cardellicchio et al. (2023) used YOLOv5 to recognize fruits, flowers and the colors of objects; Azimi et al. (2021) took advantage of deep learning (DL) to classify stress in plant shoots based on plant phenotype images; Zhou et al. (2021) applied advanced deep learning (DL) methods based on convolutional neural networks to carry out the analysis of corn phenotype. The above researches show that DL/ML has favorable potential in the classification of plant phenotype, but the obtained plant morphological traits are comparatively single and there are few studies to predict plant species and analyze insect resistance genotypes based on the morphological traits of leaves, and the related ML/DL models are highly susceptible to the influence of environment, images, data sets, etc. during the implementation of detection. In this paper, we will try to solve the above problems.

To evaluate crops based on soybean plant phenotypic information, the traditional popular machine learning (ML) often uses Shallow Neural networks, such as support vector machine (SVM), back propagation neural network (BP), generalized regression neural network (GRNN), and other models based on small datasets are often applied to construct plant gene-insect resistance models in the field of agricultural engineering (Kamilaris and Prenafeta-Boldú, 2018). Deep learning techniques, such as deep neural networks (DNN) (Du et al., 2019) , convolutional neural networks (CNN) (Cong et al., 2019) , recurrent neural networks (RNN) (Yu et al., 2019), and residual neural networks (Resnet) (Alom et al., 2019), require a large amount of data for modeling and are significantly less effective than shallow neural networks for small data (Chlingaryan et al., 2018). Owing to the difficulty of soybean phenotypic data collection, therefore, we constructed a small data set between plant phenotypes and varieties. Based on this, we used popular shallow neural networks such as Support Vector Machine (SVM), Back Propagation Neural Network (BP) and Generalized Regression Neural Network (GRNN)to build the model respectively to classify its species based on the phenotypic characteristics of soybean leaves.

Therefore, the aim of this study is to accurately extract phenotypic trait parameters from the leaves of plants with different gene expression forms of the same variety using the LPM algorithm based on the application of the SFM algorithm combined with the MVS reconstruction technique in plants. It will construct a triple linkage between genotype-phenotype-insect resistance and establish a prediction and classification model of soybean varieties. This study is organized as follows: (1) A 3D target acquisition system based on the SFM algorithm combined with MVS reconstruction technology is designed and constructed to perform 3D reconstruction of soybean plants with different gene expression forms (ko-Williams82, oe-Williams82, and Williams82) of the same variety and obtain their 3D point cloud models. (2) Point cloud data, such as individual plants, stems, and leaves, are automatically segmented using low-pass filtering, Gaussian filtering, ordinary least squares (OLS) plane fitting, and Laplacian smoothing. (3) Eleven phenotypic parameters of the leaves, including length, width, volume, projection area, projection length, tilt information and so on, are obtained using the LPM algorithm. (4) The reconstruction accuracy of the SFM-MVS algorithm is analyzed using regression evaluation indicators (RMSE, MAPE, R^2^), and the association between genotype, phenotype, and insect resistance is constructed by combining the plant penetrance parameters of different gene expression forms. (5) Three models, SVM, BP, and GRNN, are constructed to compare the prediction and classification models of soybean species based on six characteristic phenotypic parameters of leaves.

## 2 Materials and methods

### 2.1 Experimental materials and data acquisition

Three soybean varieties, ko-Williams82, oe-Williams82, and Williams82 (hereinafter referred to as C3, 47-6, and W82, respectively) were selected from the Baima Base of Nanjing Agricultural University. There were 15 plants of each variety (planted in three replicates, each in a separate row with five plants of each variety in a row), and a total of 45 soybean plant samples were collected. The soybean row spacing was 40 cm and the plant spacing was 80 cm. For the convenience of data processing in the later stage, the experimental samples were planted with potted plants (diameter of 27 cm; height of 21 cm) to avoid occlusion between plants. The soil used for soybean planting was first dried in the sun, then the dried soil was first crushed, and then the stones and weeds in the soil were removed through a 6 mm mesh screen to ensure the homogeneity of the soil. Finally, the sieved soil and nutrient soil (organic matter content >15%, total N, P, and K content >0.88%, ph7~7.5) were divided into 3:1 evenly mixed, loaded quantitatively into a plastic pot with a diameter of 30 cm, and water added to make the absolute water content of the soil 30%. Five soybean seeds were placed in each pot at a sown depth of 3.0 cm. The soybean plants were placed in a net chamber and provided normal water and fertilizer management during soybean growth. When the soybean grew to R4 stage, the density of one spot bug per plant was used for insect treatment. After 10 days of damage, dynamic non-destructive measurement and manual comparison verification of plant height, leaf length, leaf width, and other parameters of soybean plants were carried out, and the association between soybean plant genotype, phenotype, and insect resistance was established.

**Figure 1:**
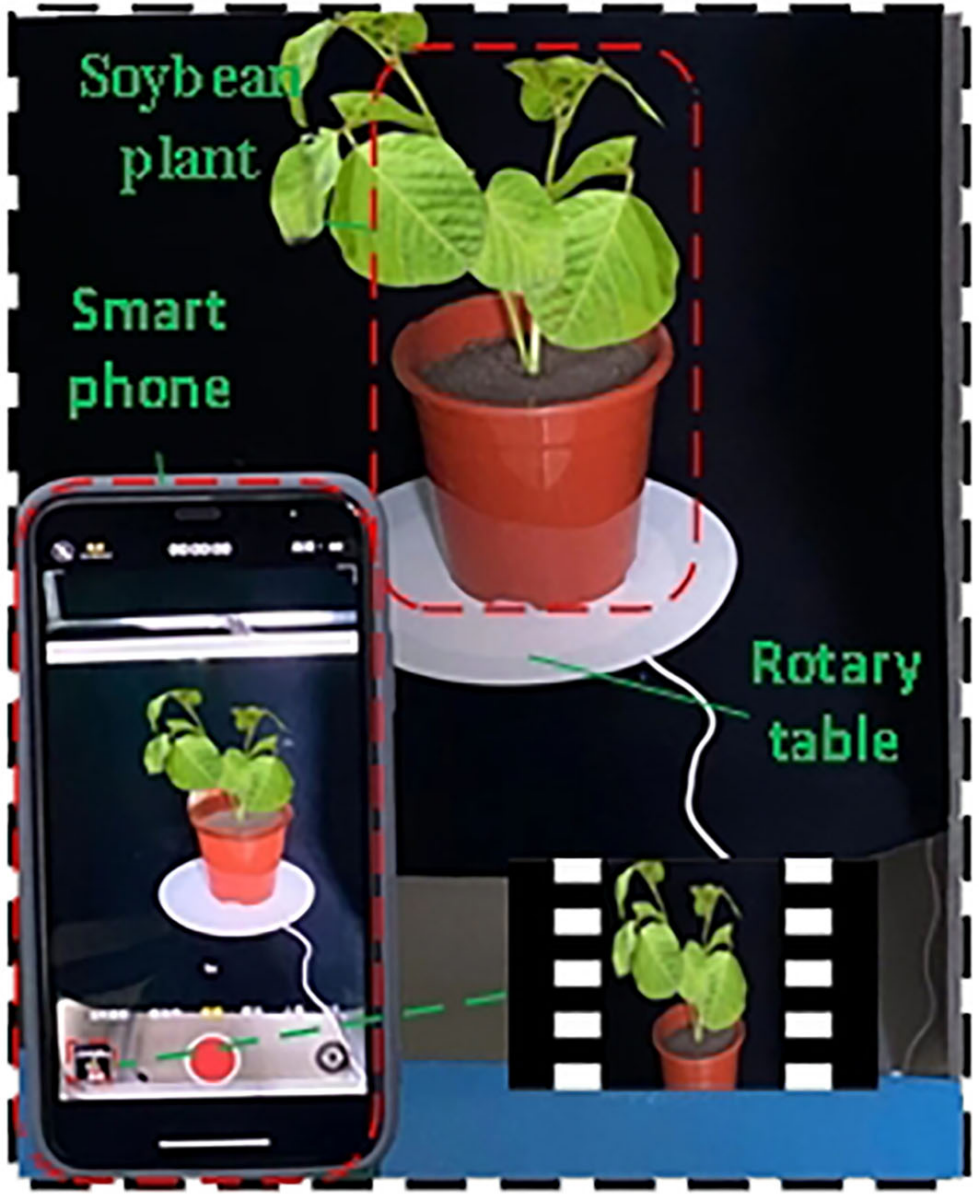
3D object acquisition platform.

A smartphone (iPhone 11) was used as the acquisition device to capture the soybean plant for 40 s. The resolution was set to 1080p HD, 60fps before video acquisition to ensure the universality of the video acquisition device. To avoid the influence of smart phone mirror shooting on 3D reconstruction, an electric turntable (diameter of 26 cm) with a speed of 0.05 r/s and a load bearing of 40 KG was used as the plant bearing platform. The smartphone was placed on a scaffold with a height of 45 cm at a distance of 25 cm from the plant, and the data at different angles of the plant were collected by tilting down 30° at a horizontal height of approximately 30 cm above the plant. The carrying platform was rotated for two weeks for video shooting, and 300 multi-view images were extracted by frame in JPG format with 1080×1920 resolution. The back and bottom of the platform were covered with a black fleece to ensure a stable and reliable recording environment and to minimize noise interference (**Figure 1**).

The specific steps of the manual measurement of soybean plant height, leaf width, and leaf length are as follows. Four workers measured the height of the same soybean plant using a scale ruler as the reference line along the basin and measured the leaf length (from leaf base to leaf tip, excluding petiole) and leaf width (the widest part on the leaf that is perpendicular to the main vein) of all the leaves of each soybean plant using a standard calculation paper with a straight ruler. The average of the readings of the four workers was taken as the final manually measured value of the phenotypic parameters of the soybean plant.

The software used for the experiment was Free Studio, the 3D reconstruction open-source software Visual SFM, and MATLAB 2022a. The electric turntable worked continuously for 40 s at a speed of 0.05 r/s to obtain the image video of the soybean plant. Three hundred multi-view images were extracted from the video obtained by frame. To ensure a large amount of accurate point cloud data, the ROI were selected from the multi-view images of the plant, and the point cloud data were generated by 3D reconstruction. The point cloud data were sampled and denoised; low-pass filtering, point cloud clustering, OLS fitting, and Laplacian smoothing were used. Parameters, such as plant height, the number of leaves, leaf length, leaf width, minimum bounding box volume of a single plant, minimum bounding box volume of a single leaf, the volume of a leaf, leaf projection area, projection length, projection width, and angle were automatically measured using the maximum traversal and greedy projection triangle algorithms. The accuracy and robustness of the SFM reconstruction of soybean plants were evaluated and compared with the manual measurement of plant height, leaf length, and leaf width.

### 2.2 Overall process of SFM-MVS method for reconstructing 3D model of soybean plants

**Figure 2:**
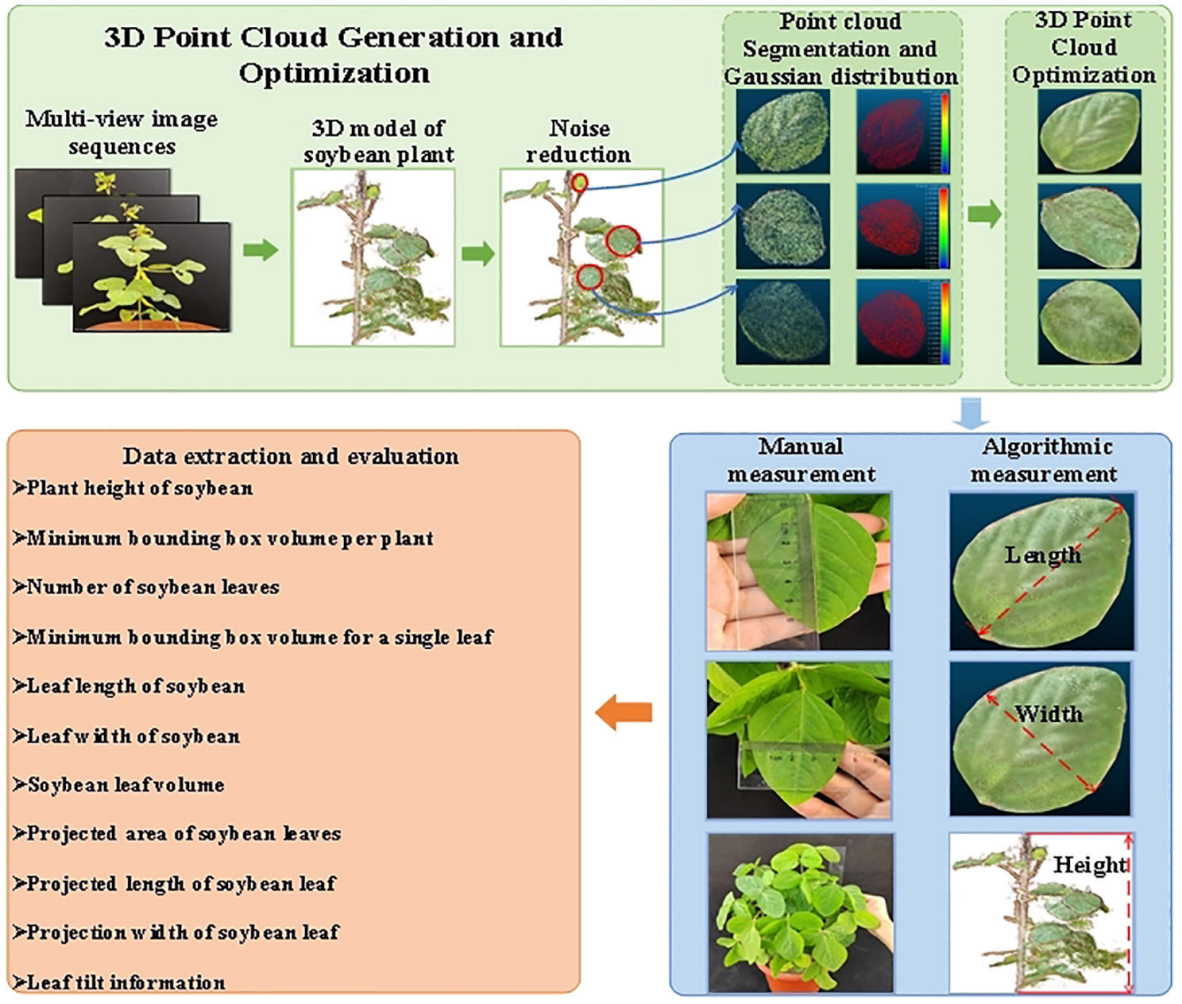
Workflow of 3D reconstruction and accuracy evaluation.

In this study, the SFM-MVS method was used to reconstruct the 3D models of soybean plants. A workflow diagram is shown in **Figure 2**. It consists of seven steps: (1) capturing multi-view images of soybean plants; (2) selecting the Plant ROI; (3) finding key points from multi-view images and reconstructing the 3D point cloud of the plant; (4) filtering and segmentation algorithms to separate leaves and stems; (5) reconstructing the smooth surface of the leaf point cloud using the plane fitting algorithm and the Laplacian smoothing algorithm; (6) extracting and evaluating plant structural phenotype parameters based on the distance maximum traversal algorithm and the greedy projection triangulation algorithm; and (7) establishing the identification of soybean varieties based on phenotypic information.

### 2.3 Extraction of ROI from soybean plants

**Figure 3:**
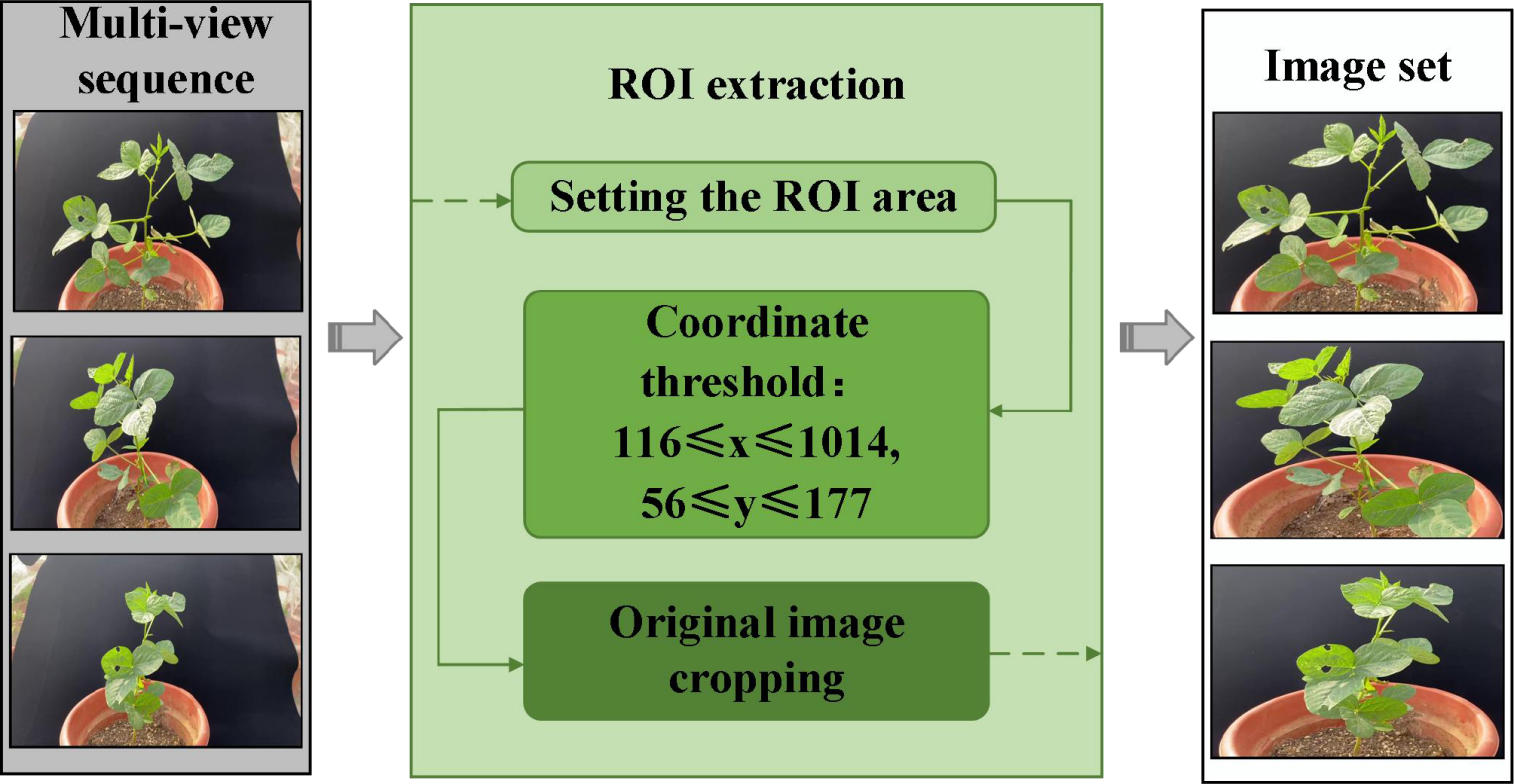
Clipping of the ROI.

This study proposes an improved detection and matching strategy to accurately obtain the key feature points of multi-view images and improve the efficiency of feature matching (**Figure 3**). The proportion of the region of interest (ROI) is increased by cropping the original image, and the scale of the image is reduced to reduce the number of calculations for feature detection.

The preliminary segmentation of soybean plant regions in multi-view images based on the ROI algorithm is a key part of the 3D reconstruction. The multi-view image sequence is cropped based on the ROI of each image, effectively reducing the resolution of the image and increasing the proportion of the soybean plant in the whole image. The rate of generation of dense point clouds was increased by 81.62% by the SFM-MVS algorithm for the 3D reconstruction of soybean plants after soybean plant ROI extraction.

### 2.4 3D model reconstruction of soybean plants

**Figure 4:**
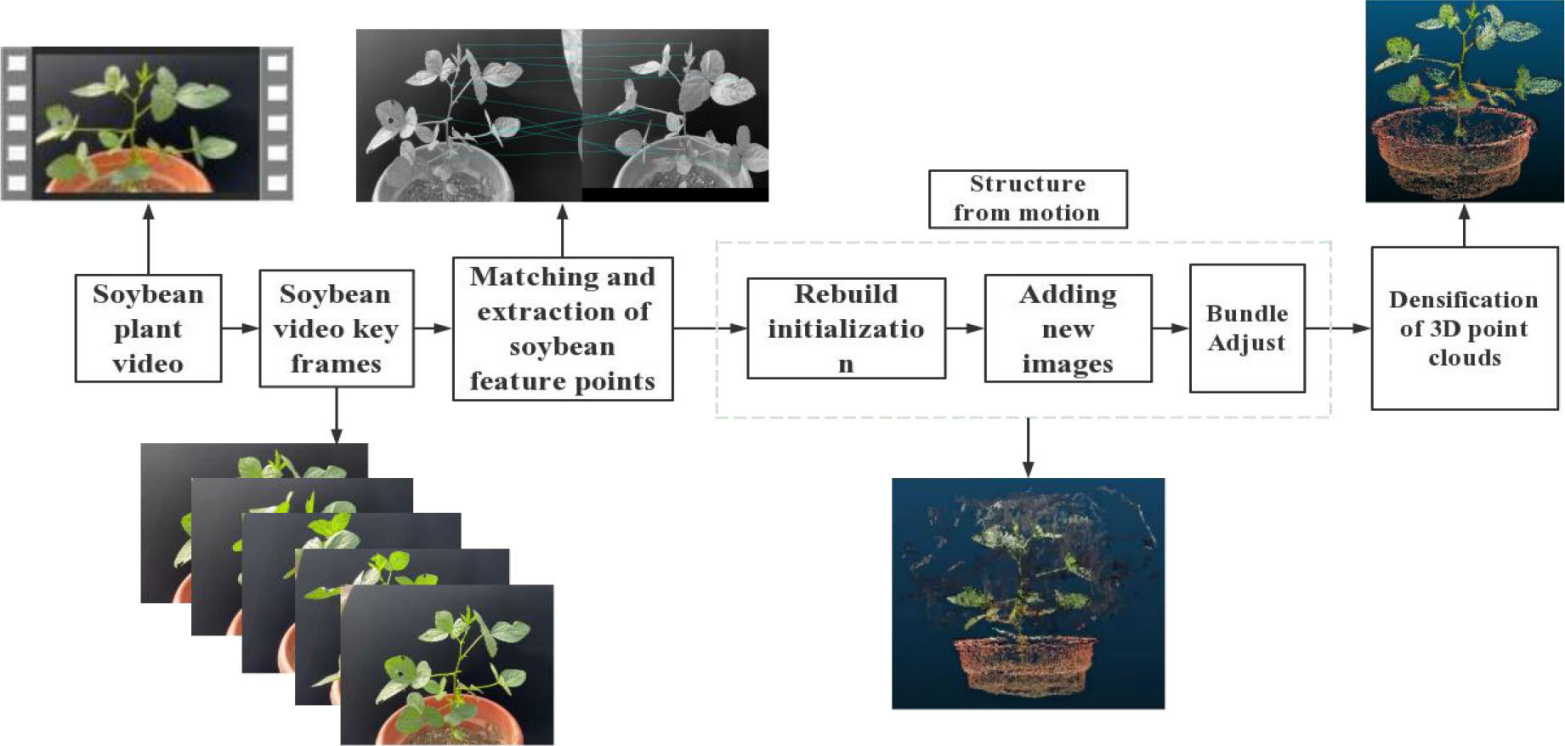
3D model reconstruction process.

We used VisualSVM software to conduct the standard sfm-mvs workflow and obtained the plant point clouds. The process of 3D model reconstruction, as shown in **Figure 4**. The main steps in soybean plant 3D model reconstruction are feature point extraction and matching, sparse point cloud reconstruction, and dense point cloud reconstruction.

### 2.5 Processing of soybean plants point cloud data

**Figure 5:**
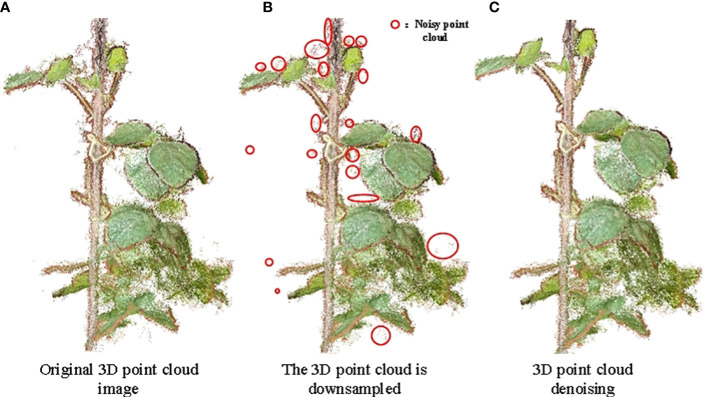
Down-sampling and denoising effect of soybean point cloud. **(A)** Original 3D point cloud image; **(B)** The 3D point cloud is downsampled; **(C)** 3D point cloud denoising.

As a result of the many dense leaves of soybean plants (**Figure 5A**), the reconstructed data were large and interspersed with a number of noisy background point clouds (**Figure 5B**). Point cloud data sampling, denoising, optimization, coordinate correction, and other processes are required because the soybean 3D point cloud model is inconsistent with the actual plant in the standard 3D space direction and scale (**Figure 5C**).

#### 2.5.1 Sampling of point cloud data

Owing to the large redundancy, long reconstruction time, and low efficiency of 3D point cloud data reconstructed using Visual SFM software, a point cloud simplification algorithm based on voxelized grid downsampling was used. Voxelized grid downsampling creates a minimum 3D voxel grid based on the point cloud data (Han et al., 2017), divides the point cloud data into a 3D voxel grid, selects a data point as the center of gravity point of the grid, and retains the data point closest to the center of gravity of the small grid. This method is simple, efficient, and does not require the establishment of a complex topological structure to simplify point cloud data, reduce operation time, and improve the program running speed (Liang et al., 2020). As shown in **Figure 5B**, the number of point clouds was reduced to 11% of that presented in **Figure 5A**, and the soybean plant phenotype did not show any change, which did not affect the extraction of its phenotypic shape parameters.

#### 2.5.2 Point cloud denoising

Owing to the influence of a series of external factors, such as data sampling equipment, external environment, and experience of experimental operators, noise points and outliers in the reconstruction process have adverse effects on trait extraction, feature matching, and surface reconstruction (Li and Cheng, 2018). A low-pass filtering algorithm was used to locally fit the soybean, and the appropriate threshold (Points/Radius was set to 0.098264, Maxerror was set to 2) was set to remove the points that deviated from the fitting plane. The background noise and most of the edge noise were removed by setting the RGB of the background (the main background noise in this study was the point cloud of the soil and basin along the color). The denoising effect of the 3D point cloud of the soybean plant is shown in **Figure 5C**, where the number of point clouds was reduced to 89% of the number of point clouds of a single plant after sampling. As shown in **Figure 5B**, the reduced points were background noise points.

#### 2.5.3 Coordinate correction of point cloud data

(1) To accurately extract the phenotypic trait parameters of soybean plants, coordinate correction is required for the 3D point cloud of soybean, and the proportional coordinates are calculated using the potted plant as the reference. The length of the potted plant in the point cloud data was calculated using the Euclidean distance algorithm and converted to obtain the transformation coefficients to obtain the true coordinates of the soybean plant. The calculation formula is as follows:


(1) (x,y,z)=α(x',y',z')

where (x,y,z)  is the length of reference in the point cloud, (x',y',z')  is the real length of reference, and α is the transformation coefficient of point cloud coordinates.

(2) The random sample consensus algorithm (RANSAC) is used to detect the ground and obtain the normal vector of the ground $\vec{m}$ , and the rotation angle θ is obtained by combining the normal vector $\vec{n}\left(0,0,1\right)\;$ of the Z-axis. The rotation matrix can be obtained by using the Rodriguez rotation formula, and the calculation formula is as follows:


(2) $$\vec{m}\cdot \vec{n}=m*n*\cos\theta$$

(3) $$\theta =\cos^{-1}\left(\frac{\vec{m}\cdot \vec{n}}{m*n}\right)$$

(4) $$R_{rot}=\vec{E}*\cos\theta +\left(\vec{m}\cdot \vec{n}\right)*\vec{d}*R\left(\theta \right)+\left(\vec{m}*\vec{n}\right)*\sin\theta$$

(5) $$R_{rot}=\left[\begin{array}{ccc} \cos\theta +d_{1}R(\theta ) & d_{1}d_{2}(R(\theta )-d_{3}\sin\theta ) & d_{2}\sin\theta +d_{1}d_{3}R(\theta )\\ d_{3}\sin\theta +d_{1}d_{2}(\theta ) & \cos\theta +d_{2}^{2}R(\theta ) & -d_{1}\sin\theta +d_{1}d_{2}R(\theta )\\ -d_{2}\sin\theta +d_{1}d_{3}R(\theta ) & d_{1}\sin\theta +d_{2}d_{3}R(\theta ) & \cos\theta +d_{3}^{2}R(\theta ) \end{array}\right]$$

where defined R(θ)=1−cosθ , respectively, m and n are respectively the lengths of $\vec{m}$ and ethe $\vec{n}$ , $\vec{E}$ is the third-order identity matrix, θ is the rotation angle, and $\vec{d}\left(d_{1},d_{2},d_{3}\right)$ is the unit vector of $\;\vec{m}*\vec{n}$ .

#### 2.5.4 Point cloud segmentation

**Figure 6:**
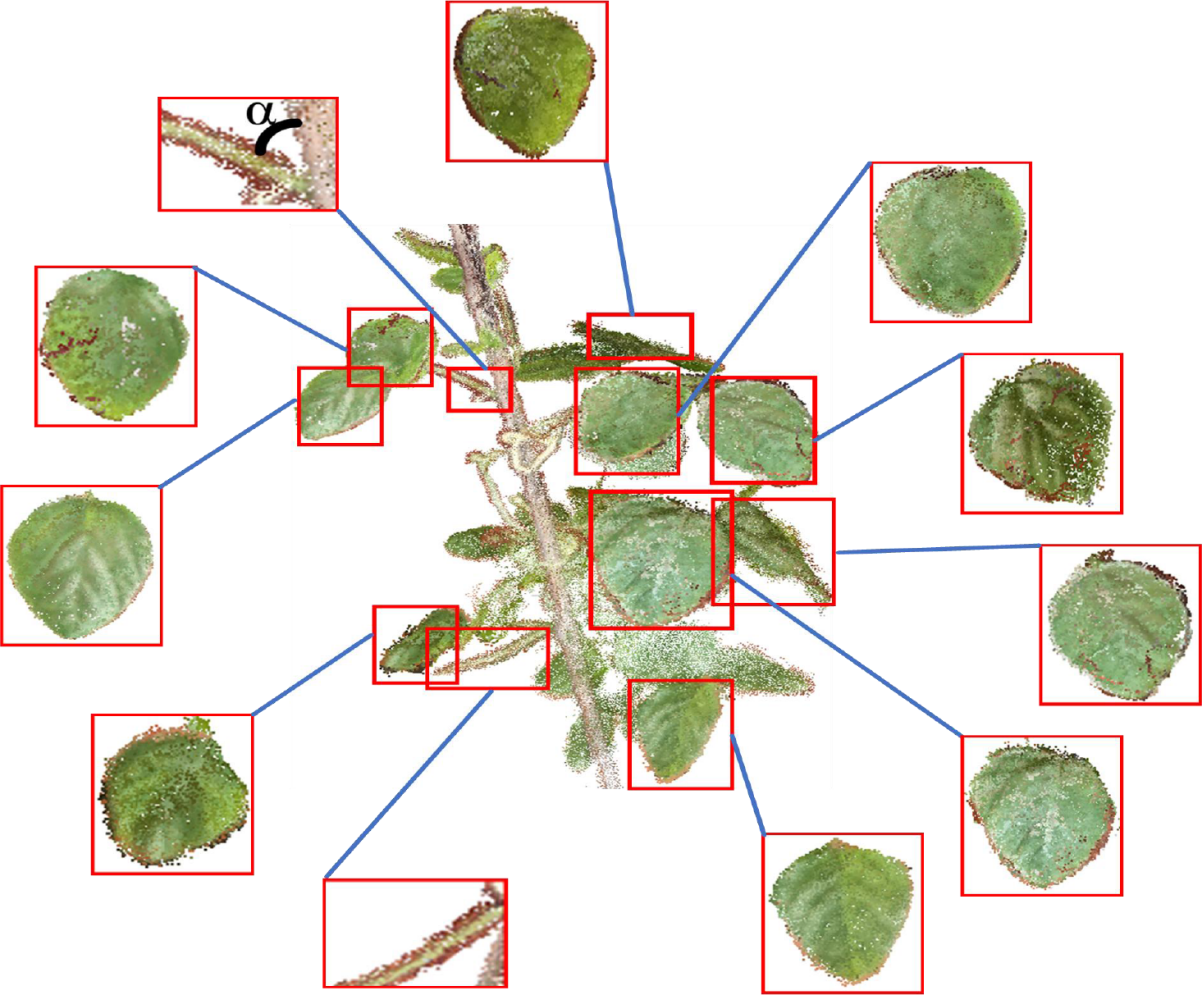
Effect of point cloud segmentation.

The 3D point cloud segmentation of soybean plants mainly aims to segment and extract the leaves and stems of soybean plants, as shown in **Figure 6**. A gap exists between any two leaves, which is a prerequisite for individual leaf segmentation. A point cloud clustering algorithm was used to segment different parts of the leaves, a cylindrical fit to the stalk of the soybean plant based on a random sampling consistency algorithm, and a statistical method to remove noise and extraneous points from the root part of the leaves was used.

#### 2.5.5 Point cloud optimization

After the point cloud segmentation of leaves and stalks, white noise generated by surface reflection or occlusion around leaves was removed based on the difference between the color of the noise and the characteristics of the leaf point cloud. The KD-Tree was used to determine the point cloud data and the distance between the fields, and the point cloud density was obtained by statistical analysis. Clutter was eliminated using the data analysis method, and the calculation formula is as follows:


(6) $$d_{i}=\sqrt{\frac{{\left(x_{ij}-x_{i}\right)}^{2}+{\left(y_{ij}-y_{i}\right)}^{2}+{\left(z_{ij}-z_{i}\right)}^{2}}{k}}$$

(7) $$\bar{d_{i}}=\frac{\sum_{i=1}^{n}d_{i}}{n}$$

(8) $$\sigma =\frac{\sum_{i=1}^{n}{\left(d_{i}-\bar{d_{i}}\right)}^{2}}{n}$$

where, $d_{i}$ is the distance between soybean point cloud and other K adjacent areas, $\bar{d_{i}}$ is the average value of the $d_{i}$ , σ standard deviation of soybean.

**Figure 7:**
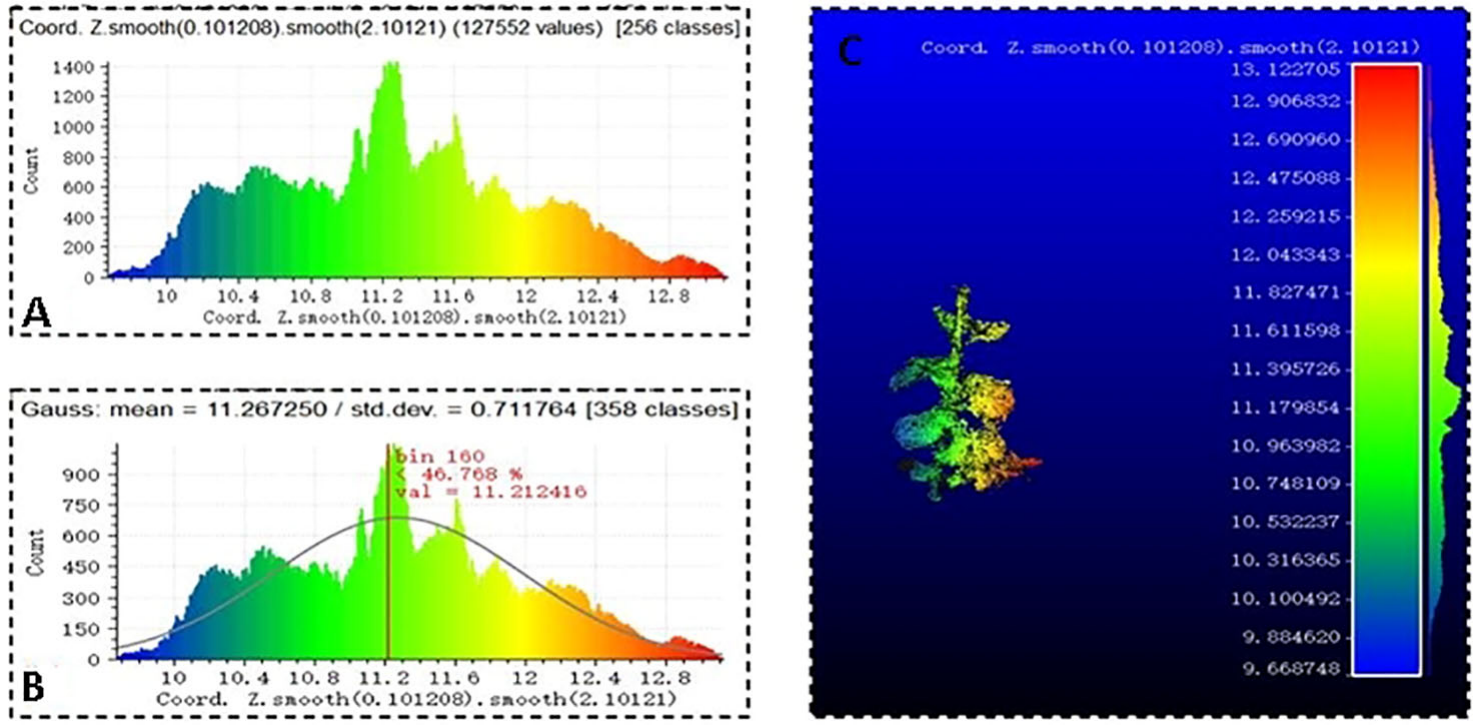
Point cloud Gaussian filtering, **(A)** soybean point cloud chromatogram, **(B)** soybean Gaussian distribution, and **(C)** filtering result.

To better realize the effect of Gaussian filtering, scalar fields were used to establish the Z-coordinate axis and draw the chromatographic diagram of the point cloud in **Figure 7A**. The Gaussian filter algorithm was used to set the covariance of the Gaussian filtering, draw the Gaussian distribution and filtering result diagram of the soybean point cloud, which are shown in **Figures 7B, C**.

The OLS plane fitting method was used to find the best matching function by minimizing the square error (Rannik et al., 2020) for the plane fitting of soybean leaves. The Laplacian smoothing algorithm was used to smooth the edges and surfaces of the soybean leaves after the initial fitting. A statistical filtering algorithm was used to optimize the soybean stalks. A 3D soybean point cloud model was obtained by splicing the optimized point cloud leaf and stem models.

### 2.6 The LPM algorithm was used to extract soybean plants traits

**Figure 8:**
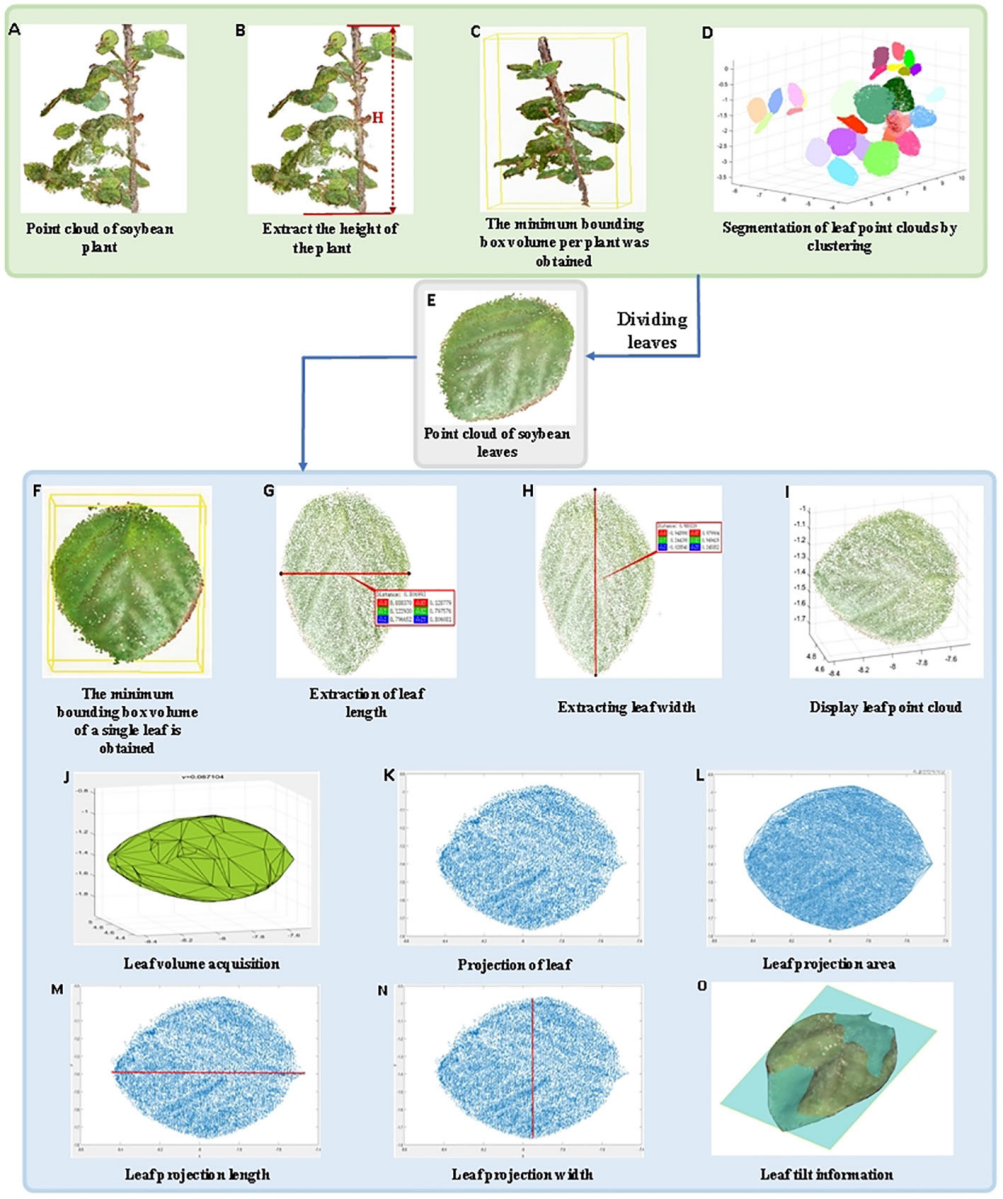
Extraction of soybean plant trait parameters. **(A)** Point cloud of soybean plant; **(B)** Extract the height of the plant; **(C)** The minimum bounding box volume per plant was obtained; **(D)** Segmentation of leaf point clouds by clustering; **(E)** Point cloud of soybean leaves; **(F)** The minimum bounding box volume of a single leaf is obtained; **(G)** Extraction of leaf length; **(H)** Extracting leaf width; **(I)** Display leaf point cloud; **(J)** Leaf volume acquisition; **(K)** Projection of leaf; **(L)** Leaf projection area; **(M)** Leaf projection length; **(N)** Leaf projection width; **(O)** Leaf tilt information.

Based on the 3D point cloud of the soybean model, the LPM algorithm is proposed in this study to calculate plant height, leaf number, length and width, minimum bounding box volume of a single plant, minimum bounding box volume of a single leaf and leaf volume, projection area, projection length, and width. The extraction process of the trait parameters is shown in **Figure 8**. First, soybean plant point cloud is displayed, the height of soybean plant and minimum volume of bounding box per plant were measured. Then, the phenotypic parameters of leaves were extracted after segmentation. The specific parameters were calculated as follows:

#### 2.6.1 Height of soybean plants

Plant height is an important indicator of plant growth in various environments (Xiao et al., 2020). The point clouds of individual soybean plants (**Figure 8A**) were extracted, and all points were traversed. After coordinate correction, the growth direction of the soybean was consistent with the z-axis direction. Therefore, the maximum value of the Z-axis coordinates between soybean and potted plants was selected, and the absolute value of the difference was the height of a single soybean plant (**Figure 8B**).

#### 2.6.2 Minimum volume of bounding box per plant

The individual soybean plants were corrected to the main direction, and the cuboid composed of yellow lines was the bounding box. The maximum x, y, and z coordinate values and the minimum x, y, and z coordinate values of the point cloud of the individual soybean plant after correction were determined, and eight vertices were obtained. The cuboid volume formed by the connection of the eight vertices corresponds to the minimum bounding box volume of the individual plant (**Figure 8C**).

#### 2.6.3 Number of soybean leaves

The non-stem point cloud was extracted to remove noise and external points, and the point cloud clustering algorithm was used to segment soybean leaves into different parts of a single plant (different colors represent different classes), where the number of different classes clustered was the number of leaves (**Figure 8D**).

#### 2.6.4 Minimum bounding box volume of a single leaf

The individual soybean plants were corrected to the main direction, and any parts of the leaves were cut (**Figure 8E**). The cuboid, which is composed of yellow lines, is the bounding box. The maximum x, y, and z coordinates and the minimum x, y, and z coordinates of the point cloud of the corrected individual soybean plants were determined, and eight vertices were obtained. The volume of the cuboid formed by the connection of these eight vertices was the minimum bounding box volume of a single plant (**Figure 8F**).

#### 2.6.5 Length of soybean leaves

The length of soybean leaves were calculated by the distance along the surface of the leaf, and any segmented leaf was extracted. The Euclidean distance algorithm was used to obtain the distance between the leaf base and leaf tip as the leaf length (**Figure 8G**).

#### 2.6.6 Width of soybean leaves

The width of soybean leaves were calculated by the distance along the surface of the leaf, and any segmented leaf was extracted. The Euclidean distance algorithm was used to obtain the maximum distance perpendicular to the leaf length as the leaf width (**Figure 8H**).

#### 2.6.7 Leaf volume of soybean

After extraction and segmentation, any soybean leaf is displayed (**Figure 8I**), and Gaussian filtering is used to de-noise the point cloud, and the envelope of its 3D point cloud is extracted. Each point cloud was divided into discrete grids, and the volume of the corresponding cell of each grid was calculated and summed to obtain the soybean leaf volume (**Figure 8J**).

#### 2.6.8 Projected area of soybean leaves

The segmented arbitrary soybean leaves were projected onto the oxy-plane, and the corresponding projected leaf point cloud was generated (**Figure 8K**). The projected leaves were triangulated using a greedy projection algorithm (Zhang Y et al., 2019), and the projected soybean leaves after triangulation were composed of small triangles. The leaf projection area of a single leaf was calculated based on the Helen formula and area summation formula (**Figure 8L**). The formula used is given by


(9) $$S_{i}=\sqrt{p_{j}\left(p_{i}-a_{j}\right)\left(p_{j}-b_{j}\right)\left(p_{j}-c_{j}\right)}$$

(10) $$S_{2D}=\sum_{j=0}^{m}S_{j}$$

where, $p_{j}$ is half of the perimeter of the triangulated triangle, $a_{j},b_{j}$ and $c_{j}$ are the lengths of each side of the triangulated triangle, m is the total number of triangulated triangles, j is the index number of triangulated triangles, $S_{j}\;$ is the projection area of a single planar triangulated facet, and $S_{2D}$ is the total projection area of a single leaf.

#### 2.6.9 Projection length of soybean leaves

The segmented soybean leaves were projected onto the oxy plane to generate the corresponding projected leaf point cloud, and the maximum and minimum values of the length-direction coordinates were calculated. The absolute value of the difference was the default length of the soybean leaf projections (**Figure 8M**).

#### 2.6.10 Projection width of soybean leaves

The segmented soybean leaves were projected onto the oxy plane to generate the corresponding projected leaf point cloud, and the maximum and minimum values of the width-direction coordinates were calculated. The absolute value of the difference was the default width of the soybean leaf projections (**Figure 8N**).

#### 2.6.11 Tilt information of leaves

The growth situation and environmental problems of soybeans can be determined based on the tilt information of soybean leaves. RANSAC plane fitting was used to obtain the plane, fitting variance RMSE, and tilt matrix, which can judge the tilt direction from a series of point cloud information using an iterative method (**Figure 8O**).

### 2.7 Modeling based on plant phenotype prediction

In this study, for three soybean varieties (C3, 47-6, W82) in R4 stage, because it is difficult to obtain the information of leaves and only a small data set is available, we used popular shallow neural networks such Support Vector Machine (SVM), Back Propagation Neural Network (BP) and Generalized Regression Neural Network (GRNN) to construct the model and select the optimal one.

Support Vector Machine (SVM) (Deng et al., 2019) is based on statistical theory and its learning model algorithm, which determines the optimal classification hyperplane in the high-dimensional feature space of data by solving optimization problems. The least-squares support vector machine (LS-SVM) overcomes the computational burden of its constrained optimization programming based on SVM to handle complex data classification more effectively.

Back Propagation Neural Network (BP) (Ju and Feng, 2019) neural network is a multi-layer feedforward network trained by an error backpropagation algorithm. The phenotypic data of plant leaves were used as the input of the BP neural network, and the output was the predicted value of the plant varieties.

Generalized Regression Neural Network (GRNN) (Dai et al., 2019) has strong nonlinear mapping ability and learning speed. In terms of classification and fitting, the GRNN model performed better when the accuracy of the plant phenotypic parameter data was poor.

Since model prediction was made based on leaf morphological traits and the light source maps the leaf vertically, the data of leaf length and width are highly similar to the data of leaf projection length and width. Therefore, Six experimental parameters (minimum bounding box volume of a single leaf, leaf volume, projection length of soybean leaves, projection width of soybean leaves, projected area of soybean leaves and leaf tilt information) are preferably selected. The input datatype for training (e.g., X is (447 x 6) array that records 6 traits of 447 leaves, Y is (447 x 1) array that records the cultivars of corresponding, use integer as labels) to construct the models of soybean sample variety prediction. For each prediction model, 80% samples are randomly selected as the training set and 20% samples are used as the test set to detect the prediction effect.

### 2.8 Accuracy evaluation

The soybean plant height, leaf length, and leaf width measured by the algorithm were compared with manual measurement values to evaluate the accuracy of the proposed method. The accuracy was measured using the mean absolute percentage error (MAPE), root mean square error (RMSE), and determination coefficient (R^2^) to evaluate the accuracy of the SFM algorithm. Correlation coefficients of calibration (Rc)、Root mean square error of calibration (RMSEC)、Correlation coefficients of prediction (Rp) and Root mean square error of prediction (RMSEP) are often used for evaluating the accuracy of models.

Mean absolute percentage error (MAPE) (Chen et al., 2020) is often used to evaluate the prediction of performance, which intuitively reflects the difference between the real value and the predicted value, usually in the range up to 100%. Root mean square error (RMSE) (Hodson, 2022) is used to measure the deviation between the predicted value and true value, and is more sensitive to outliers in the data. Determination coefficient (R2) (Piepho, 2019) is an important statistic that reflects the goodness of fit of the model. The value ranges from 0 to 1, and closer to 1 means better; Correlation coefficients of calibration (Rc) (Wang et al., 2020) as the correlation coefficient of determination for calibration, commonly used to evaluate model results, and with the value closer to 1 being better; Root mean square error of calibration (RMSEC) (Hacisalihoglu et al., 2022) is often used as an evaluation of quantitative models; Correlation coefficients of prediction (Rp) (Wang et al., 2020) as the correlation coefficient of determination for the prediction set, with the value closer to 1 means better; Root mean square error of prediction (RMSEP) (Cominotte et al., 2020) is commonly used to verify the prediction error of the model internally or externally, and is the most critical parameter for evaluating the goodness of a model.

## 3 Results

### 3.1 Results and analysis of LPM algorithm

**Figure 9:**
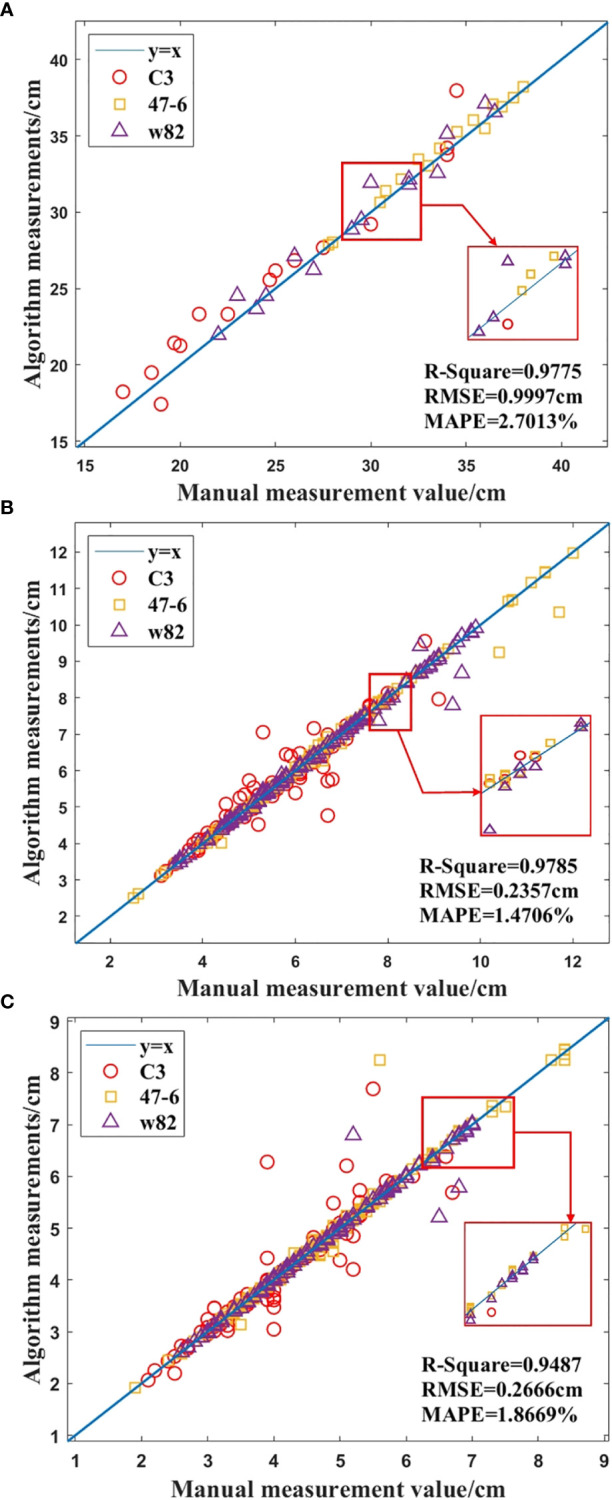
Comparison of manual and algorithmic measurements of soybean plant traits, **(A)** Height of the plant, **(B)** Length of the leaf, **(C)** Width of the leaf.

In this study, a total of 45 soybean samples from three soybean varieties (C3, 47-6, W82) in the R4 stage were used for 3D reconstruction using the SFM algorithm, and the plant height and leaf point clouds of soybean plants were automatically segmented, measured, and analyzed. In the 3D point cloud of the soybean plant, the plant trait parameters measured by the algorithm were proportionally converted, and the automatically measured plant height, leaf length, and leaf width were compared with the manually measured values. **Figure 9** shows the results.

As shown in **Figure 9A**, R^2^=0.9775, MAPE = 2.7013%, RMSE = 0.9997 cm, and the accuracy of the plant height measurement by the algorithm was 97.2987%. In addition, R^2^=0.9785, MAPE = 1.4706%, and RMSE = 0.2357 cm, and the accuracy of the leaf length measurement was 98.5294%, as shown in **Figure 9B**. As shown in **Figure 9C**, R^2^ = 0.9487, MAPE= 1.8669%, and RMSE = 0.2666 cm, and the accuracy of leaf width measurement by the algorithm was 98.1331%. According to **Figure 9**, the results show that the proposed method has high accuracy, and the algorithm measurements are in good agreement with human measurements.

### 3.2 Prediction results of plant varieties

In this study, three modeling methods, such as BP, SVM, and GRNN were used to establish soybean plant variety prediction models. Soybean leaf phenotypic parameters and the soybean plant variety were used as model inputs and the output, respectively. Among them, RMSEC is often used as an evaluation of quantitative models; RMSEP is often used to validate the prediction error of a model internally or externally; Rc as the correlation coefficient of determination for calibration; Rp is used as the correlation coefficient of determination of the prediction set. The modeling results based on the six leaf phenotypic parameters are listed in **Table 1**.

**Table 1:** Modeling results of leaf phenotypic parameters.

Model	Rc	RMSEC	Rp	RMSEP
LS-SVM	0.6934	0.5979	0.6536	0.6995
BPNN	0.7781	0.6419	0.5716	0.9528
GRNN	0.9744	18.3263	0.9211	18.9024

By modeling the leaf phenotypic parameters in **Table 1** to predict the types of soybean plants, the GRNN model had the highest prediction accuracy. The training set Rc of soybean plants was 0.9744, and the prediction set Rp was 0.9211.

## 4 Discussion

Zareef et al. (2019) used Partial Least Squares Regression (PLSR) based on the phenolic compounds of Congo black tea to predict and construct the model. The prediction accuracy of Gallic acid was 0.9111, and the prediction accuracy of Rutin was 0.8255; Hasan et al. (2021) applied six commonly ML methods (SVM, Adaboost, Logistic Regression, etc.), the gene models of Roaceae, rice and Arabidopsis were predicted and constructed, and the prediction accuracy was 0.918,0.827,0.635, respectively; Yoosefzadeh-Najafabadi et al. (2021) took advantage of three common ML (MLP, SVM, RF) based on hyperspectral reflectance data to predict and construct a soybean seed yield model, and the accuracy of the model was 0.87. The above methods use multiple models to classify and predict the phenotypes and compounds of multiple experimental objects quickly and efficiently, but the accuracy is relatively low.

The LPM algorithm used in this paper is combined with GRNN to construct a soybean prediction model, and the accuracy of model can reach 0.9211. In the paper, the 3D model of soybean plant can be reconstructed quickly and accurately by using motion restoration structure algorithm and multi-view stereo vision algorithm; The LPM algorithm can effectively measure the phenotypic parameters of 11 plant three-dimensional models, and constructed the relationship between phenotype and insect resistance; The optimal model GRNN was established to accurately predict and identify plant varieties based on the morphological traits of leaves.

**Figure 10:**
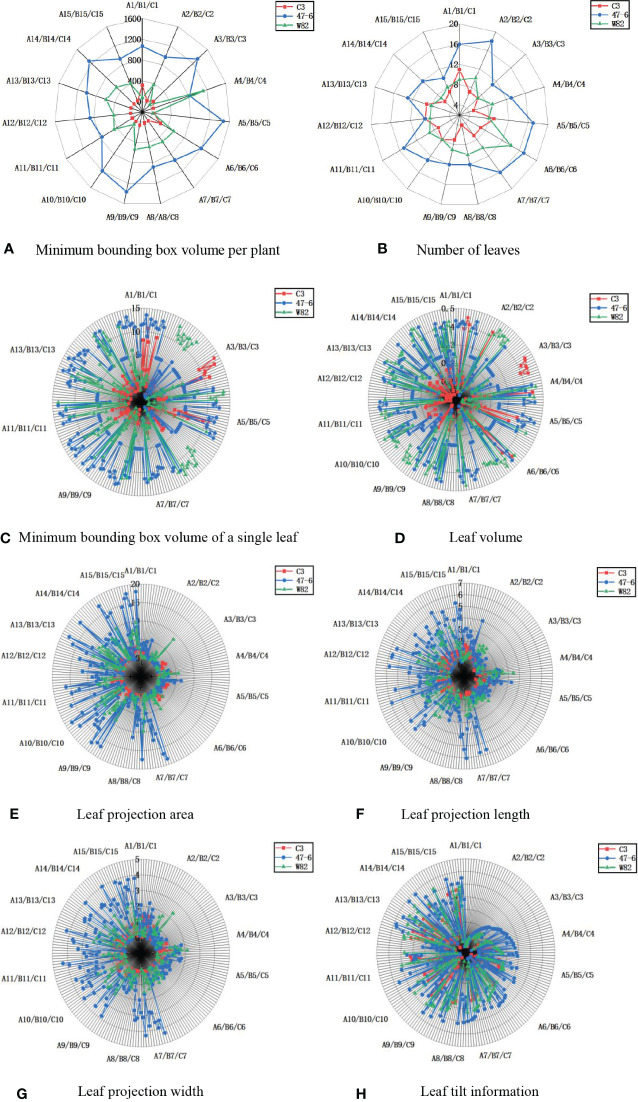
Measurement results of soybean plant trait parameters, **(A)** Minimum bounding box volume per plant, **(B)** Number of leaves, **(C)** Minimum bounding box volume of a single leaf, **(D)** Leaf volume, **(E)** Leaf projection area, **(F)** Leaf projection length, **(G)** Leaf projection width, **(H)** Leaf tilt information.

In terms of individual plant character parameters (minimum bounding box volume per plant, number of leaves, minimum bounding box volume per leaf, leaf volume, leaf projection area, leaf projection width, leaf projection length, and leaf tilt information), the soybeans of the C3 variety were lower than that of the 47-6 and W82 varieties, as shown in **Figure 10**. Soybean plant variety 47-6 were higher than soybean of variety W82 in terms of four trait parameters (minimum enclosing box volume per plant, number of leaves, leaf projected width, and leaf projected area). Soybean of varieties 47-6 and W82 were higher than soybean of variety W82 in four trait parameters (minimum enclosing box volume per plant, number of leaves, minimum enclosing box volume per leaf, and leaf projection area). There were no highly significant differences between the 47-6 and W82 varieties in terms of four trait parameters (leaf projection length, leaf volume, leaf projection width, and leaf tilt information).

C3, 47-6, and W82 are different gene expression forms of the same variety, where 47-6 (oe-Williams82) is a certain gene overexpression strain and C3 (ko-Williams82) is a gene knockout strain. Differences in gene expression may be the reason for the changes in the overall parameters, and the differences in gene expression will lead to changes in the surface hairs of the soybean. These hairs of soybean pods of the 47-6 overexpressed variety were sparse, and the pods were easily fed on by stink bugs. The stink bugs bite the soybean pods through the mouth, resulting in the normal development of soybean seeds (Chen et al., 2018) and the formation of aborted seeds. Here, the sink and source relationship is confusing. Therefore, the plant will use more nutrients to promote the vegetative growth and growth of its node, make the plant taller, and increase the volume of the minimum bounding box per plant and the number of leaves. However, pod feeding of *M. obstatus* did not affect changes in leaf morphology-related information, such as leaf projection length, leaf volume, leaf projection width, and leaf tilt information. C3 is an insect-resistant line, which is considerably slightly damaged by the bug. Thus, the trait parameters of C3 are significantly less than 47-6, and gene knockout affects the changes in leaf morphology-related information parameters. Plant phenotypic traits can be divided into physiological, morphological, and component traits (Danilevicz et al., 2022). Among the three major targets of breeding, such as the yield, quality, and resistance, the resistance target (biotic stress or abiotic stress) is particularly important and indicates the core productivity to ensure stable yield. Among them, changes in morphological and structural traits, such as plant height and leaf area, are the most intuitive reflections of plant resistance and they play an important role in the study of insect resistance (Nelson et al., 2018).

## 5 Conclusion

The soybean plant 3D structure was successfully obtained by SfM, and a good correction (R2>0.94) and small RMSE (<0.24) were observed with manual measured. Compared to SVM and BPNN, the GRNN showed the highest accuracy (0.9211) of the cultivar classification tasks.

In this paper, we mainly focus on the 3D reconstruction of soybean plants (ko-Williams82, oe-Williams82, and Williams82), and analyze the relationship between phenotypic traits and insect resistance genes. In the later stage, a whole set of machines will be developed to expand the number of soybean varieties and monitor the growth changes of soybean plants in real-time to further enhance the practicability and realize more comparisons of soybeans between species and genotypes to select superior insect-resistant varieties.

## Data availability statement

The raw data supporting the conclusions of this article will be made available by the authors, without undue reservation.

## Author contributions

WH proposed the conceptualization and methodology, and wrote the paper. ZY programmed the software. ML compared the performance of the algorithms. YY designed and carried out the experiments. WL and GX improved the methodology and conceived the experiments. All authors reviewed the manuscript. All authors contributed to the article and approved the submitted version.
